# Longitudinal 16S rRNA data derived from limb regenerative tissue samples of axolotl *Ambystoma mexicanum*

**DOI:** 10.1038/s41597-019-0077-7

**Published:** 2019-05-23

**Authors:** Turan Demircan, Ayşe Elif İlhan, Guvanch Ovezmyradov, Gürkan Öztürk, Süleyman Yıldırım

**Affiliations:** 1 Department of Medical Biology, International School of Medicine, İstanbul Medipol University, İstanbul, Turkey; 2 Department of Biostatistics and Medical Informatics, International School of Medicine, Istanbul Medipol University, Istanbul, Turkey; 3 Department of Physiology, International School of Medicine, İstanbul Medipol University, İstanbul, Turkey; 4 Department of Microbiology, International School of Medicine, İstanbul Medipol University, Istanbul, Turkey; 5 Regenerative and Restorative Medicine Research Center, REMER, İstanbul Medipol University, İstanbul, Turkey

**Keywords:** Regeneration, Conservation biology, Microbiome, RNA sequencing

## Abstract

The Mexican axolotl (*Ambystoma mexicanum*) is a critically endangered species and a fruitful amphibian model for regenerative biology. Despite growing body of research on the cellular and molecular biology of axolotl limb regeneration, microbiological aspects of this process remain poorly understood. Here, we describe bacterial 16S rRNA amplicon dataset derived from axolotl limb tissue samples in the course of limb regeneration. The raw data was obtained by sequencing V3–V4 region of 16S rRNA gene and comprised 14,569,756 paired-end raw reads generated from 21 samples. Initial data analysis using DADA2 pipeline resulted in amplicon sequence variant (ASV) table containing a total of ca. 5.9 million chimera-removed, high-quality reads and a median of 296,971 reads per sample. The data constitute a useful resource for the research on the microbiological aspects of axolotl limb regeneration and will also broadly facilitate comparative studies in the developmental and conservation biology of this critically endangered species.

## Abstract (summary)

**Table:** 

Design Type(s)	time series design • organism development design
Measurement Type(s)	rRNA 16S
Technology Type(s)	DNA sequencing
Factor Type(s)	biological replicate • temporal_interval
Sample Characteristic(s)	Ambystoma mexicanum • limb • laboratory environment

Machine-accessible metadata file describing the reported data (ISA-Tab format)

## Background & Summary

Urodele amphibians are promising vertebrate model organisms to study regeneration due to their astonishing capacity of tissue repair and renewal. Of these amphibians, Mexican axolotl (*Ambystoma mexicanum*), a critically endangered species^1^, is one of the few adult animals capable of complete and functional regeneration of missing body parts throughout its life^2^. Axolotl can regenerate their extremities including limbs^3^, tail^4^, brain^5^, spinal cord^6^ and internal organs^7^ during larval and adult stages. Despite growing publicly available resources for cellular and molecular research on axolotl regeneration^8,9^ (e.g. transcriptome^3,10–14^, proteome^15,16^, and genome data^17^), the axolotl microbiome data remain scarcely available, which hinders comprehensive interpretation of the accumulating data in this field. As an initial effort to bridge this knowledge gap, we recently reported first multi-organ microbiome profile of axolotl at neotenic and metamorphic stages^18^. However, longitudinal profiling of microbial communities in the course of axolotl limb regeneration has not been reported before. Here, we describe bacterial 16S rRNA amplicon datasets derived from axolotl limb tissue samples in the course of limb regeneration.

**Fig. 1:**
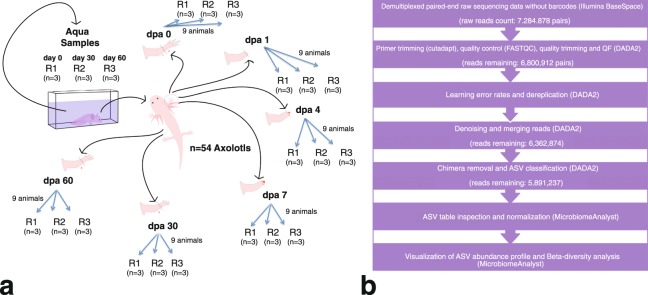
Experimental design. (**a**) The datasets generated in these experiments were derived from 21 samples in total (18 limb tissue samples and 3 aquarium water samples). Post amputation limb tissue samples were collected at these experimental timepoints: 0 dpa, 1 dpa, 4 dpa, 7 dpa, 30 dpa and 60 dpa; “dpa” denoting day post amputation. At each experimental time point groups included 3 biological replicates (R1, R2 and R3) and each replicate was formed by pooling limb tissue samples from 3 animals to minimize interindividual variation. The aquarium water samples were collected at 3 time points (day 0, day 30 and day 60) in 3 replicates (R1, R2, and R3). (**b**) The workflow of data analysis, the employed tools and changes in the number of processed reads at each step are shown.

**Fig. 2:**
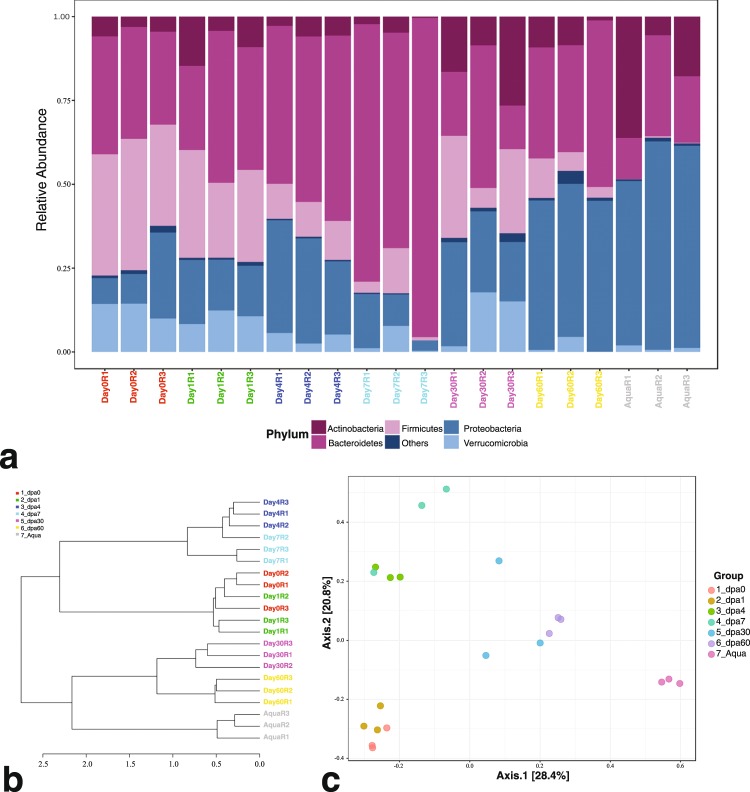
Structure and diversity of bacterial communities associated with regenerating axolotl limb tissues. (**a**) Stacked bar chart of shows shifts in relative bacterial abundance on phylum level in the course axolotl limb regeneration. The microbial profile demonstrates temporal dynamics with the underlying differential phyla abundances in this biological process and depicts separation of aquarium control groups. (**b**) Hierarchical clustering of samples based on Beta-diversity analysis of ASV abundances. This chart was generated using Bray-Curtis distance metric and Ward’s method (as linkage method). (**c**) Principal Coordinate Analysis (PCoA) of all studies samples based on Beta-diversity analysis of ASV abundances. This chart was generated using Bray-Curtis distance metric and PCoA ordination method. The Fig. 2b,c shows clustering of treatment samples into three main groups (corresponding to the three main stages of axolotl limb regeneration, namely wound healing, dedifferentiation and re-development) and their separation from the control group.

The study design aimed to profile the microbiome of limb tissues around the cut site at different stages of regeneration (0, 1, 4, 7, 30, and 60 days post-amputation, “dpa”) by sequencing 16S rRNA gene amplicons. Sampling site and time points from intact or regenerating limbs are depicted in Fig. 1a. The experimental sample collection time points corresponded to three main phases of axolotl limb regeneration, namely initiation phase (dpa 0 and dpa 1), early phase (dpa 4 and dpa 7) and late phase (dpa 30 and dpa 60). These sequential phases encompass wound healing, dedifferentiation (highlighted by blastema establishment) and re-development stages of regeneration, respectively^19^. The raw sequencing data comprised 14,569,756 paired-end reads generated from 21 samples. The data analysis workflow is shown in Fig. 1b. We employed DADA2 pipeline and generated an ASV abundance table containing ca. 5.9 million chimera-removed, high-quality reads and a median of 296,971 reads per sample (range: 129,428–409,656). The phyla *Bacteroidetes*, *Firmicutes*, *Proteobacteria*, *Actinobacteria* and *Verrucomicrobia* (Fig. 2a) dominated the bacterial communities. In addition, the obtained microbial community profile demonstrated lower intra-group variation when compared to the inter-group variation (Fig. 2a). Based on beta-diversity analyses, microbial communities of limb tissues at regeneration initiation phase, early regeneration phase, and redevelopment phase samples clearly separated (Fig. 2b,c, see methods section), indicating temporal shifts in bacterial composition between sampled tissues. Finally, aquarium water sample controls clustered separately from axolotl tissue samples (Fig. 2b,c), suggesting water microbiota colonization in axolotl limb tissues is minimal.

To date only few studies have examined the role and importance of microbiome in tissue regeneration in animals^20,21^. The datasets described here offer a valuable new resource for this emerging area of research and underscore the value of the axolotl as a model organism for regeneration biology. We anticipate that our data will broadly contribute to comparative and/or correlative studies employing multi-omics techniques in the developmental, regenerative, and conservation biology of amphibians.

## Methods

### Ethical statement

Experimental protocols and animal care conditions were approved by the local ethics committee of the Istanbul Medipol University (IMU) with authorization number 38828770-433.

### Animal husbandry and experimental design

In this study, 54 adults (1-year-old, 12–15 cm in length) axolotls chosen randomly among siblings were included. Founders of initial axolotl colony was purchased from Kentucky, USA and maintained and bred in animal care facility of the IMU in keeping with the established protocols^22^. The randomly picked experimental animals were then housed separately as one individual in a cuboid shaped aquarium filled with Holtfreter’s solution at 18 ± 2 °C temperature and maintained at this temperature throughout the experiments. The animals were fed once a day with a staple food (JBL Novo LotlM, Neuhofen, Germany). The experimental design is depicted in Fig. 1a. In each group, 9 animals were randomly sub-grouped into three biological replicates (R1, R2 and R3) to assess the replicability of the results. During the experimental period all animals were maintained as one animal per aquaria. To amputate axolotl limbs, we first anesthetized the animals using 0.1% Tricaine methane sulfonate (Cat. No. E10521 or MS-222, Sigma-Aldrich, St. Louis, MO, USA) then amputated the right forelimb of each animal at mid-zeugopod level. Following the amputation, we randomly selected the amputated animals to form six groups representing three main phases of axolotl limb regeneration, the initiation phase (dpa 0 and dpa 1), the early phase (dpa 4 and dpa 7) and the late phase (dpa 30 and dpa 60). To minimize the variation between individuals, tissue samples from three animals were pooled together for each biological replicate. All tissue samples were cryopreserved in liquid nitrogen immediately after the collection and stored at −80 °C until genomic DNA isolation. Dpa 0 and dpa 1 samples were isolated from approximately 1-mm tissue around the cut site. Dpa 4 and dpa 7 were isolated by removing the newly formed blastema and 0.5 mm posterior tissue from the cut site since 0.5 mm posterior tissue of cut site was previously reported to be important zone for dedifferentiation of cells into stem/progenitor cells^23^. To sample the microbiota of the restored tissues in the newly formed limbs, dpa 30 and dpa 60 samples were again collected around the original cut site.

To investigate whether microbiota in Holtfreter’s solution colonized axolotl limbs, we collected water samples (100 ml) from a total of 9 individual aquaria in the course of experimental timeline. We pooled water samples from aquaria of 3 axolotls at the beginning (day0-R1), 3 water samples in the middle (day30-R2), and 3 at the end (day60-R3) and named these samples the “aqua” control group, resulting 3 replicates (R1, R2, R3; see Fig. 1a).

### DNA extraction, PCR and 16S rRNA amplicon sequencing

DNeasy Blood & Tissue Kit (Qiagen, Cat No. 69504) was used to isolate genomic DNA from the collected samples by following the manufacturer’s instructions. DNA of water samples was extracted by using ‘Metagenomic DNA Isolation Kit for Water’ (Epicentre, Cat. No. MGD08420). Concentrations of the isolated genomic DNA samples were determined using Qubit 2.0 Fluorometer (Thermo Fisher Scientific, MA, USA) and dsDNA BR Assay Kit (ThermoFisher Cat. No. Q32850). Integrity of the DNA samples were ascertained by running an aliquot of the DNA samples on a 1.0% agarose gel. Primers 337F (5′-GACTCCTACGGGAGGCWGCAG-3′) and 806R (5′-GGACTACHVGGGTWTCTAAT-3′), with tails of Nextera adapter sequences were used to amplify the targeted V3–V4 region of the 16S rRNA gene, as described in the illumina 16S rRNA metagenomic sequencing protocol.

For each sample, PCR was carried out in total volume of 25 µl reaction mixture which included 12.5 ng of purified DNA template and 2x KAPA HiFi HotStart Ready Mix. The following order of steps and conditions was used for PCR: initial denaturation at 95 °C for 3 minutes and 25 cycles of; denaturation at 95 °C for 30 seconds, annealing at 55 °C for 30 seconds and extension at 72 °C for 30 seconds, with a final extension at 72 °C for 5 minutes. The PCR products were purified by using Agencourt AMPure XP purification system (Beckman Coulter, Cat. No. A63881, USA) and second PCR (with 8 cycles) was performed by using sample-specific barcodes and the obtained amplicons were purified again. Equimolar concentrations from each library were then pooled and sequenced on Illumina MiSeq sequencer using MiSeq Reagent Kit v2, 500 cycles. The obtained sequences were on average 231 bp long. The raw data resulting from this sequencing effort can be found in NCBI Sequence Read Archive^24^. Accession numbers, sample source and experimental time point from each sample can be found in Table 1.

**Table 1:** Meta table of samples in the dataset.

Sample name	Biosample accession	Isolation source	Isolation time	DNA source	Library preperation	Library description	Library layout	Instrument model
Day0R1	SAMN09722311	limb	dpa 0	Whole Community DNA	PCR	16S rRNA amplicon sequencing	paired	Illumina Miseq
Day0R2	SAMN09722312	limb	dpa 0	Whole Community DNA	PCR	16S rRNA amplicon sequencing	paired	Illumina Miseq
Day0R3	SAMN09722313	limb	dpa 0	Whole Community DNA	PCR	16S rRNA amplicon sequencing	paired	Illumina Miseq
Day1R1	SAMN09722314	limb	dpa 1	Whole Community DNA	PCR	16S rRNA amplicon sequencing	paired	Illumina Miseq
Day1R2	SAMN09722315	limb	dpa 1	Whole Community DNA	PCR	16S rRNA amplicon sequencing	paired	Illumina Miseq
Day1R3	SAMN09722316	limb	dpa 1	Whole Community DNA	PCR	16S rRNA amplicon sequencing	paired	Illumina Miseq
Day4R1	SAMN09722317	limb	dpa 4	Whole Community DNA	PCR	16S rRNA amplicon sequencing	paired	Illumina Miseq
Day4R2	SAMN09722318	limb	dpa 4	Whole Community DNA	PCR	16S rRNA amplicon sequencing	paired	Illumina Miseq
Day4R3	SAMN09722319	limb	dpa 4	Whole Community DNA	PCR	16S rRNA amplicon sequencing	paired	Illumina Miseq
Day7R1	SAMN09722320	limb	dpa 7	Whole Community DNA	PCR	16S rRNA amplicon sequencing	paired	Illumina Miseq
Day7R2	SAMN09722321	limb	dpa 7	Whole Community DNA	PCR	16S rRNA amplicon sequencing	paired	Illumina Miseq
Day7R3	SAMN09722322	limb	dpa 7	Whole Community DNA	PCR	16S rRNA amplicon sequencing	paired	Illumina Miseq
Day30R1	SAMN09722323	limb	dpa 30	Whole Community DNA	PCR	16S rRNA amplicon sequencing	paired	Illumina Miseq
Day30R2	SAMN09722324	limb	dpa 30	Whole Community DNA	PCR	16S rRNA amplicon sequencing	paired	Illumina Miseq
Day30R3	SAMN09722325	limb	dpa 30	Whole Community DNA	PCR	16S rRNA amplicon sequencing	paired	Illumina Miseq
Day60R1	SAMN09722326	limb	dpa 60	Whole Community DNA	PCR	16S rRNA amplicon sequencing	paired	Illumina Miseq
Day60R2	SAMN09722327	limb	dpa 60	Whole Community DNA	PCR	16S rRNA amplicon sequencing	paired	Illumina Miseq
Day60R3	SAMN09722328	limb	dpa 60	Whole Community DNA	PCR	16S rRNA amplicon sequencing	paired	Illumina Miseq
AquaR1	SAMN09722329	aquarium	day 0	Whole Community DNA	PCR	16S rRNA amplicon sequencing	paired	Illumina Miseq
AquaR2	SAMN09722330	aquarium	day 30	Whole Community DNA	PCR	16S rRNA amplicon sequencing	paired	Illumina Miseq
AquaR3	SAMN09722331	aquarium	day 60	Whole Community DNA	PCR	16S rRNA amplicon sequencing	paired	Illumina Miseq

### Data analysis

The overview of the data analysis workflow is shown in Fig. 1b. Demultiplexing and clipping of sequence adapters and barcodes from raw sequences were performed using the Illumina BaseSpace platform (Illumina). The resulting 16S rRNA paired-end raw sequencing data comprised in total ca. 14.6 million reads generated from 21 samples. We then employed cutadapt program^25^ v1.13 (default parameters) to remove the primers preceding 16S rRNA amplicon reads. The primer-removed raw sequences were subsequently uploaded to the online Nephele analysis platform v2 together with the mapping file^26^, which describes the individual raw data files to otherwise entirely automatic online pipeline. The FastQC software^27^, which is part of the Nephele Pre-processing Quality Check (QC) pipeline, was used to check the quality of the Illumina sequencing reads before the reads were fed into the Nephele DADA2 pipeline (which implements DADA2 package, v1.6^28^). The logfiles generated by the two pipelines^26^ describe each step in data processing.

The DADA2 software integrated to Nephele DADA2 pipeline automatically performed quality check prior to data analysis. Briefly, after quality check (Supplementary Figs 1 and 2), the pipeline performs quality trimming and filtering, dereplicates sequences, learns error rates (Supplementary Figs 3 and 4), removes sequences potentially containing errors (denoising), merges paired-end reads as contigs, screens contigs for mismatches to reduce errors, constructs amplicon sequence variant (ASV) abundance table, removes chimeric sequences (using “bimera” method), runs taxonomic classification of ASVs using SILVA reference (SILVA 132 release) database^29^. In the quality trimming and filtering step, “truncation quality score equal to 4 (truncQ = 4)” parameter was selected and reads with maximum expected errors greater than 5 were discarded as a quality filtering measure (using “maxEE = 5” parameter). The pipeline detected 7.4% of all reads (in terms of relative abundance) as chimeric (“bimera”) and removed from datasets. The resulting ASV table^26^ thereby retained high quality nonchimeric reads (5.9 million reads in total generated from 21 samples, with the median 296,842 reads per sample).

Sequencing depth of raw data and number of reads per sample remaining after each data analysis step are shown in Table 2. Rarefaction plot for the ASV abundance dataset ensured sufficient depth for sample comparison (Supplementary Fig. 5). Downstream analysis included data inspection, normalization, abundance visualization and beta-diversity analysis steps. This part of the workflow was performed using the freely available web-based tool, “MicrobiomeAnalyst”^30^, specifically the Marker Data Profiling (MDP) module integrated into this webtool. After initial data filtering step 786 ASVs were used for the subsequent analyses. The normalization step was performed to account for compositional differences by applying rarefaction to the minimum library size and data scaling based on the Total Sum Scaling (TSS) method. The normalized ASV abundance data was used for visualization of ASV relative abundances and beta-diversity analysis. Relative phylum-level abundances were shown in stacked barplot (Fig. 2a). Beta-diversity of bacterial communities in the limb samples were shown using hierarchical clustering (Ward’s linkage method) (Fig. 2b) and the Principal coordinate analysis (PCoA) based on Bray-Curtis index^31^ (Fig. 2c) and tested using Permutational multivariate analysis of variance (PERMANOVA) (F-value: 8.4063; R-squared: 0.78274; *p*-value < 0.001), Homogeneity of Group Dispersions (PERMDISP)^32^ (F-value: 1.5975; *p*-value: 0.21993), and Analysis of Group Similarities (ANOSIM) (R: 0.90577; *p*-value < 0.001).

**Table 2:** Data analysis steps and statistics of sequencing read counts.

Sample name	Biosample accession	Raw reads count (2x, paired end)	After QF	After denoising and merging reads	After chimera removal and ASV classification	% loss compared to raw reads count
Day0R1	SAMN09722311	342,852	321,854	305,502	294,755	14.03
Day0R2	SAMN09722312	351,732	328,845	315,912	309,042	12.14
Day0R3	SAMN09722313	406,113	364,723	342,772	330,958	18.51
Day1R1	SAMN09722314	195,403	168,877	149,490	144,943	25.82
Day1R2	SAMN09722315	427,304	407,498	386,102	360,187	15.71
Day1R3	SAMN09722316	310,119	293,501	283,956	278,140	10.31
Day4R1	SAMN09722317	278,646	261,178	249,804	242,528	12.96
Day4R2	SAMN09722318	355,593	334,229	320,922	315,410	11.30
Day4R3	SAMN09722319	357,377	341,935	326,755	311,928	12.72
Day7R1	SAMN09722320	391,963	375,936	362,778	348,131	11.18
Day7R2	SAMN09722321	344,771	328,160	317,877	313,793	8.99
Day7R3	SAMN09722322	438,949	426,027	417,232	409,656	6.67
Day30R1	SAMN09722323	363,335	331,968	308,732	296,971	18.27
Day30R2	SAMN09722324	365,793	340,974	323,572	320,444	12.40
Day30R3	SAMN09722325	348,330	323,147	305,834	294,501	15.45
Day60R1	SAMN09722326	280,446	256,267	240,793	234,231	16.48
Day60R2	SAMN09722327	446,357	417,638	395,121	388,892	12.87
Day60R3	SAMN09722328	295,612	272,966	253,801	248,840	15.82
AquaR1	SAMN09722329	289,438	261,743	224,997	129,428	55.28
AquaR2	SAMN09722330	331,887	306,163	250,180	155,313	53.20
AquaR3	SAMN09722331	362,858	337,283	280,742	163,146	55.04
SUM		7,284,878	6,800,912	6,362,874	5,891,237	19.13

## Data Records

The 16S rRNA gene sequencing raw data (paired-end reads) were deposited in the NCBI Sequence Read Archive^24^ under the BioProject ID PRJNA482947 with the SRA accession number SRP155412. The deposited dataset consists of 42 compressed FASTQ paired-end files (forward and reverse reads), generated from 21 samples. The supplementary files can be found within the Figshare repository^26^.

## Technical Validation

Our unique experimental design (Fig. 1a) enabled us to assess both biological and technical variations. Of the limb tissues we collected we pooled 9 biological tissue samples into 3 biological replicates to reduce and assess within and between group variations. We also ensured to include negative controls in genomic DNA extractions and no template controls in all PCR runs; no detectable bands were observed on agarose gel electrophoresis from these controls. Furthermore, aquarium water samples were used as control group to assess the profile of microbiota in axolotl’s aqueous environment.

Quality profiles generated by the quality control step based on Phred quality scores (Supplementary Figs 1 and 2) and the learning error rates step of the Nephele DADA2 pipeline (Supplementary Figs 3 and 4) assisted in optimizing the quality trimming and filtering. The recently developed ASV-based approach was preferred over the traditional OTU-based approaches for assessing microbiome abundance since this novel approach has been shown to generate more precise and accurate results for analysis of 16S rRNA data^28,33,34^. As an ASV-based method, the novelty and resulting improved resolution of the DADA2 package performance comes especially from the dereplication and denoising functionalities^28^.

Finally, to further demonstrate usability and replicability of the datasets described here, we included 16S rRNA sequencing data from limb skin samples, which we collected in our previous study^18^. The obtained results indicated that skin samples from our previous study were very similar to the regeneration initiation phase samples (Supplementary Figs 6 and 7).

## Supplementary Information

### ISA-Tab metadata file

Download metadata file

### Supplementary information

Supplementary Figures

## Data Availability

No custom codes were used in generating or processing of the dataset described herein.
