# Supplementary material for: Longitudinal 16S rRNA data derived from limb regenerative tissue samples of axolotl Ambystoma mexicanum
Source: Sci Data. 2019 May 23;6:70. doi: 10.1038/s41597-019-0077-7 (PMC6533342; doi:10.1038/s41597-019-0077-7)
Supplement: Supplementary file 2 — Supplementary Figures [file 41597_2019_77_MOESM2_ESM.pdf]

SUPPLEMENTARY FIGURES

Longitudinal 16S rRNA data derived from limb regenerative tissue samples of axolotl *Ambystoma mexicanum*

Turan Demircan[1,5*], Ayşe Elif İlhan[5], Guvanch Ovezmyradov[2,5], Gürkan Öztürk[3,5], Süleyman Yıldırım[4,5*]

Description	Page
**Supplementary Figure 1**	**2**
**Supplementary Figure 2**	**3**
**Supplementary Figure 3**	**4**
**Supplementary Figure 4**	**5**
**Supplementary Figure 5**	**6**
**Supplementary Figure 6**	**7**
**Supplementary Figure 7**	**8**

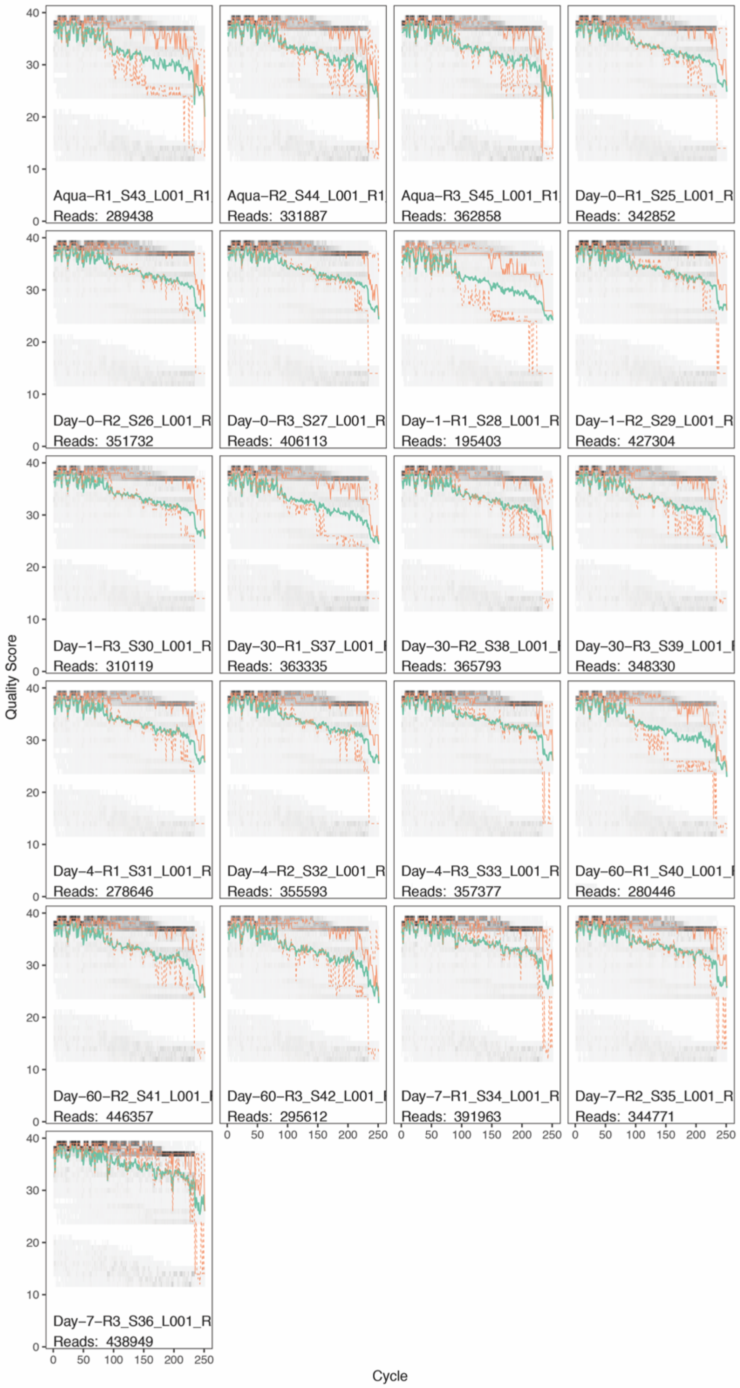

Supplementary Figure 1. Quality profiles based on Phred quality scores for forward sequencing reads. This visualization summarizes the quality score distribution in reference to each base position in sequences (denoted as "Cycle"). On these plots, the bases are along the x-axis and the quality score on the y-axis. Respective read counts for each forward input FASTQ file are also shown. A grey-scale heatmap displays the quality score distribution at each read position, with the black underlying heatmap shows the frequency of each score at each base position. Positional summary statistics is visualized using the plotted lines: green line, orange line and the dashed orange lines represent the mean, the median and the 25th and 75th quantiles, respectively.

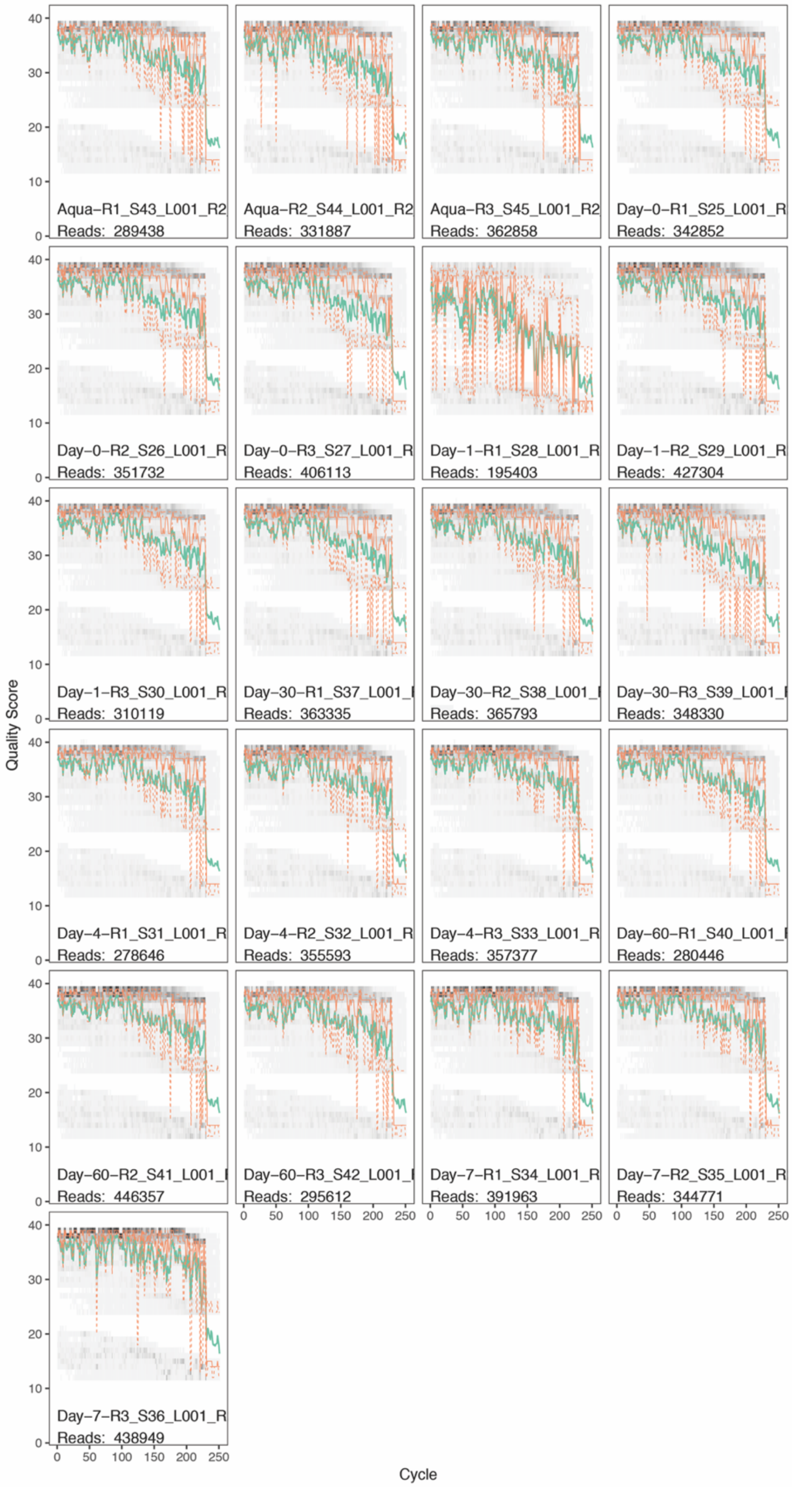

Supplementary Figure 2. Quality profiles based on Phred quality scores for reverse sequencing reads. This visualization summarizes the quality score distribution in reference to each base position in sequences (denoted as "Cycle"). On these plots, the bases are along the x-axis and the quality score on the y-axis. Respective read counts for each reverse input FASTQ file are also shown. A grey-scale heatmap displays the quality score distribution at each read position, with the black underlying heatmap shows the frequency of each score at each base position. Positional summary statistics is visualized using the plotted lines: green line, orange line and the dashed orange lines represent the mean, the median and the 25th and 75th quantiles, respectively.

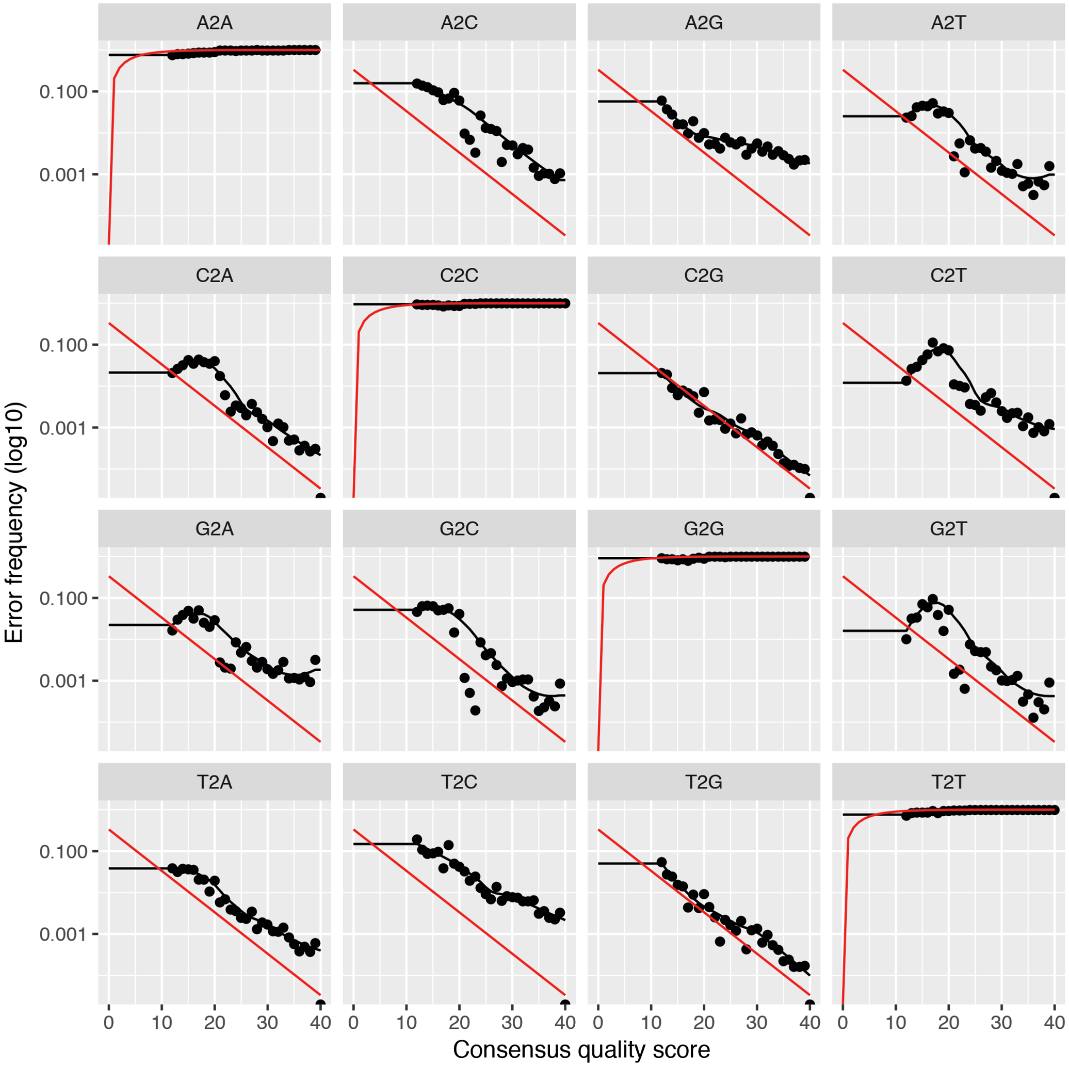

Supplementary Figure 3. The charts show error rates of the dataset learned using DADA2 program for forward reads. The error rates are plotted as error frequency associated with each possible transition (for example, T to A) with regard to the related consensus quality score. Observed error rates are depicted as black points. Estimated error rates were learned by the DADA2 program and are plotted as the black line. The expected error rates are based on the nominal definition of the Phred quality scores and are plotted as the red line.

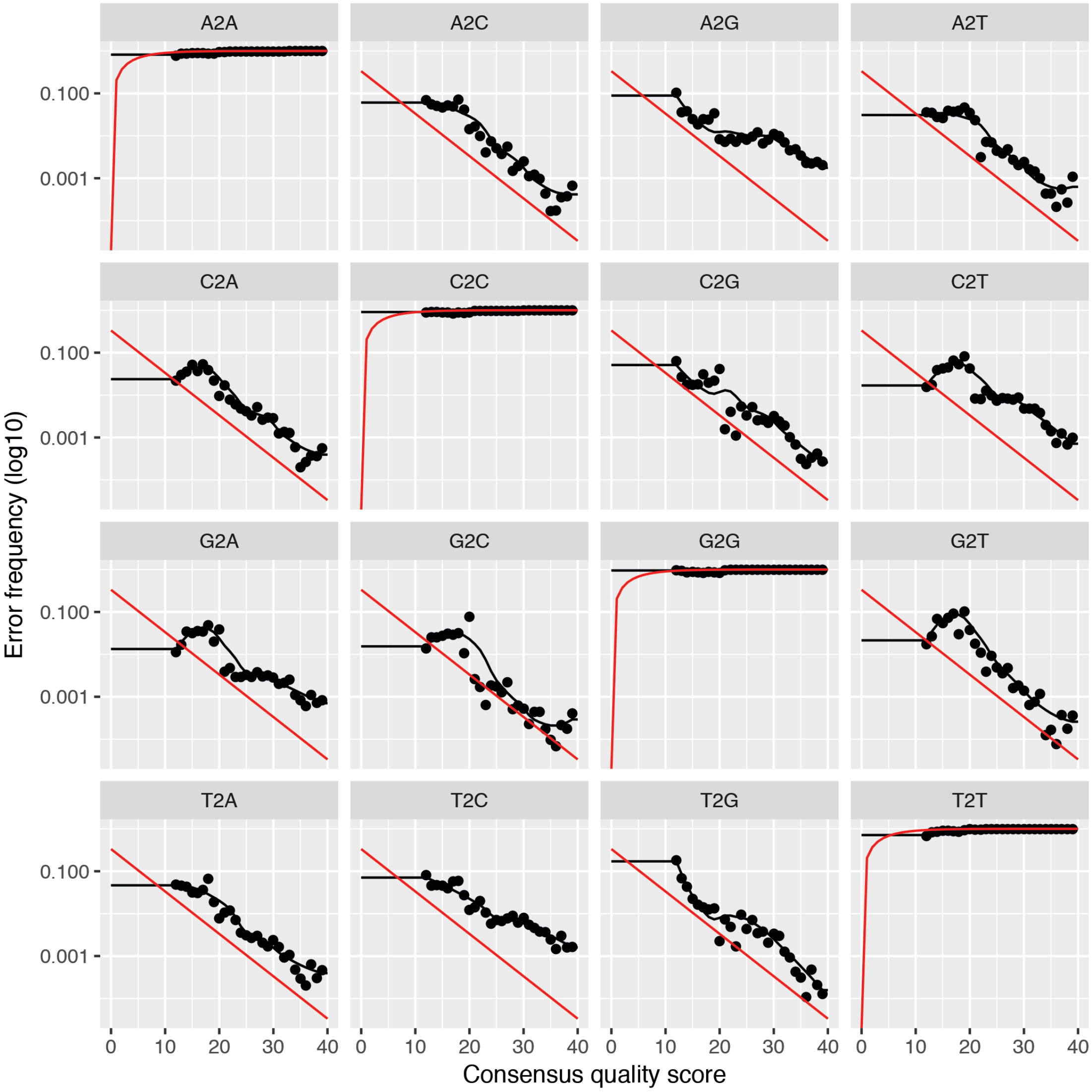

Supplementary Figure 4. Error rates of the dataset learned using DADA2 program for reverse reads. The error rates are plotted as error frequency associated with each possible transition (for example, T to A) with regard to the related consensus quality score. Observed error rates are depicted as black points. Estimated error rates were learned by the DADA2 program and are plotted as the black line. The expected error rates are based on the nominal definition of the Phred quality scores and are plotted as the red line

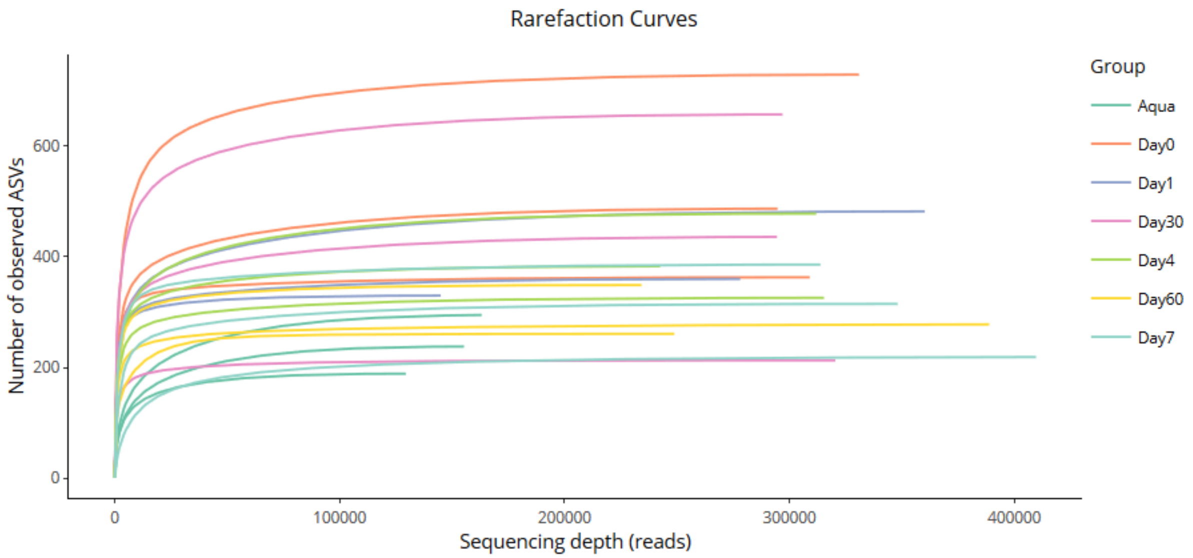

Supplementary Figure 5. Rarefaction plot illustrating sequencing depth with regard to the number of observed ASVs.

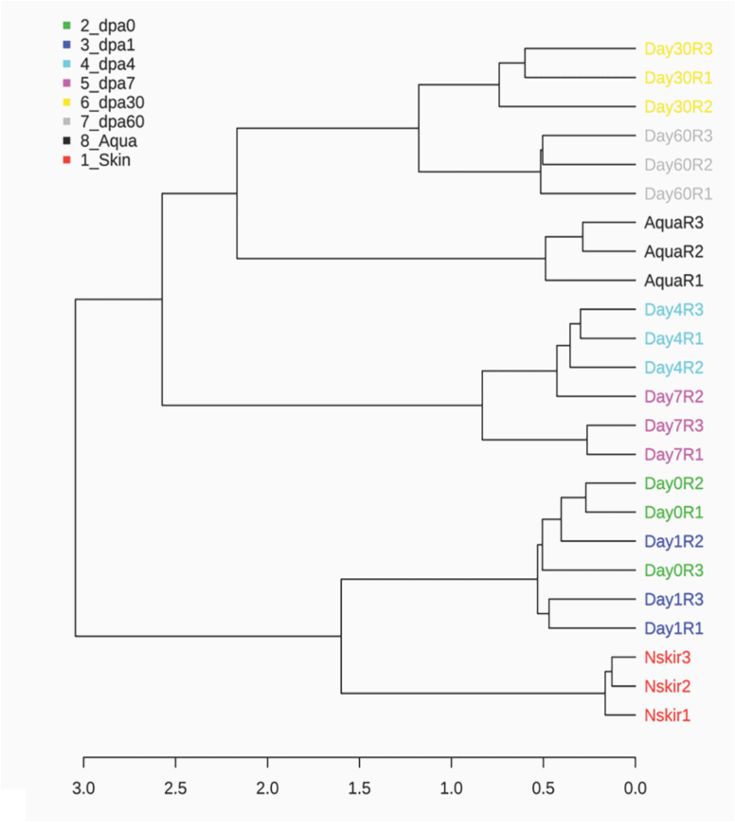

Supplementary Figure 6. Structure and diversity of bacterial communities associated with regenerating axolotl limb tissues and untreated (normal) skin. Dendrogram using Ward's Linkage method showing cluster relationship between samples based on Bray-Curtis distance metric.

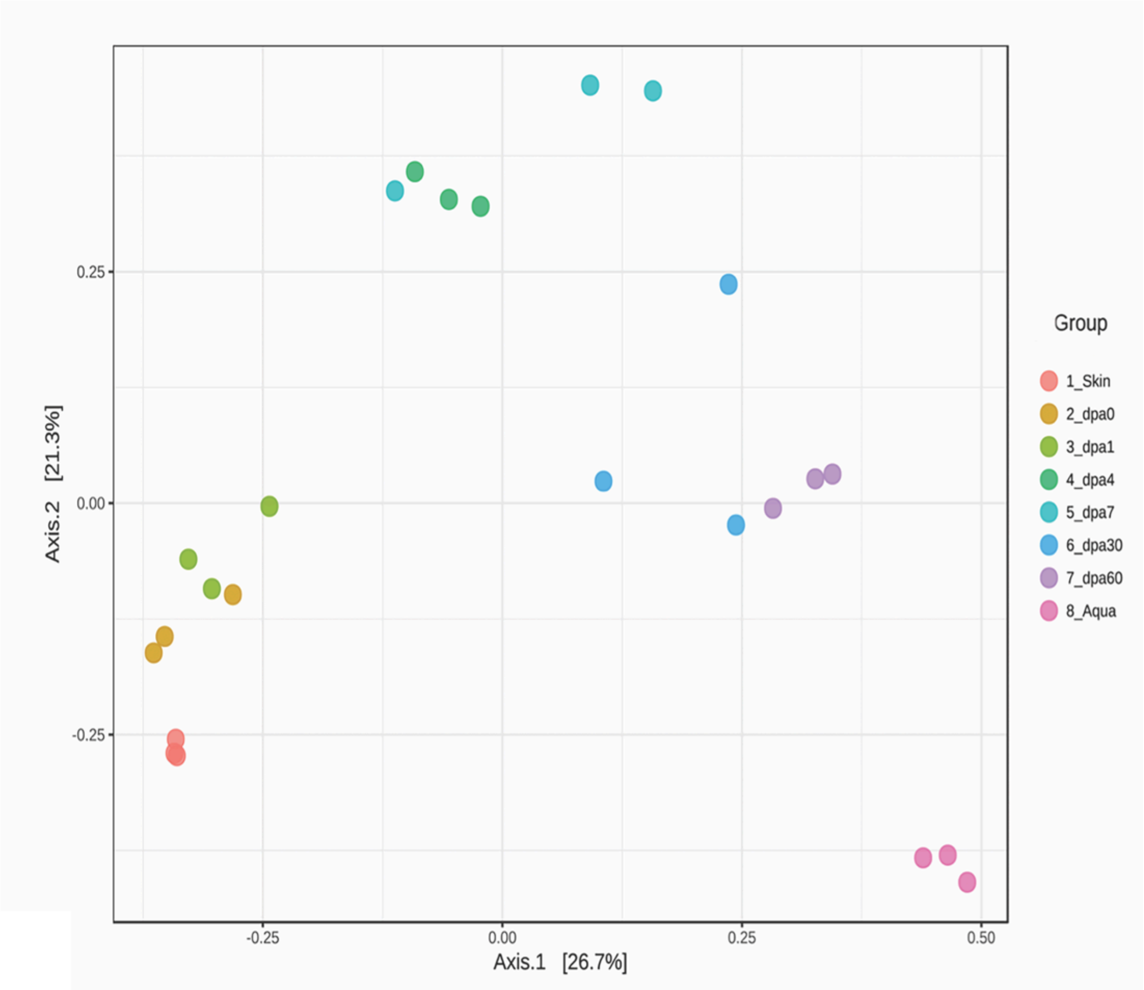

Supplementary Figure 7. Structure and diversity of bacterial communities associated with regenerating axolotl limb tissues and untreated (normal) skin. Principal Coordinate Analysis (PCoA) of all samples based on Bray-Curtis distance. The figure shows clustering of treatment samples and similarity between untreated (normal) neotenic skin samples from the previously published study (Demircan et. al.,2018) and dpa0 and dpa1 samples in this study

Reference cited:

Demircan, T. *et al.* Experimentally induced metamorphosis in highly regenerative axolotl (*Ambystoma mexicanum*) under constant diet restructures microbiota. *Scientific Reports* **8**, 10974 (2018).
